# Attenuation of Upper Body Accelerations during Gait: Piloting an Innovative Assessment Tool for Parkinson's Disease

**DOI:** 10.1155/2015/865873

**Published:** 2015-10-11

**Authors:** Christopher Buckley, Brook Galna, Lynn Rochester, Claudia Mazzà

**Affiliations:** ^1^MRC-Arthritis Research UK Centre for Integrated Research into Musculoskeletal Ageing (CIMA), Pam Liversidge Building, University of Sheffield, Sheffield S1 3JD, UK; ^2^Department of Mechanical Engineering, University of Sheffield, Sir Frederick Mappin Building, Sheffield S1 3JD, UK; ^3^Institute of Neuroscience/Newcastle University Institute for Ageing, Newcastle University, Clinical Ageing Research Unit, Campus for Ageing and Vitality, Newcastle upon Tyne NE4 5PL, UK; ^4^INSIGNEO Institute for in Silico Medicine, University of Sheffield, Pam Liversidge Building, Sheffield S1 3JD, UK

## Abstract

The objective of the current investigation was to explore whether upper body accelerations obtained during gait provide sensitive measures of postural control in people with Parkinson's disease (PD). Thirteen people with PD (70 ± 11 years) and nineteen age-matched controls (70 ± 7 years) walked continuously for two minutes while wearing three inertial sensors located on their lower back (L5), shoulder level (C7), and head. Magnitude (root mean square (RMS)), attenuation (attenuation coefficient), and smoothness (Harmonic ratios, HR) of the accelerations were calculated. People with PD demonstrated greater RMS, particularly in the mediolateral direction, but similar harmonic ratio of head accelerations compared to controls. In addition, they did not attenuate accelerations through the trunk and neck as well as control participants. Our findings indicate that measuring upper body movement provides unique information regarding postural control in PD and that poor attenuation of acceleration from the pelvis to the head contributes to impaired head control. This information is simple to measure and appears to be sensitive to PD and, consequently, is proposed to benefit researchers and clinicians.

## 1. Introduction

People with Parkinson's disease (PD) walk with a gait pattern that is characterised by slowness (bradykinesia), muscle rigidity, and postural instability [1–3]. As the disease progresses, postural control deteriorates and predisposes people with PD to falls [4–6]. Current measures of postural control, based on the ability to maintain upright balance during quiet stance, poorly reflect real life situations when people with PD are at risk of falling. Consequently, researchers and clinicians are promoting the measurement of postural control during gait [7, 8].

The recent development of small and inexpensive wireless inertial sensors has helped facilitate routine measurement of postural control during gait in the clinic, laboratory, and the community. Emerging evidence suggests that measuring upper body acceleration during gait using inertial sensors can objectively quantify differences in gait patterns between those with and without PD [9, 10]. It has also been shown that upper body accelerations are sensitive to differences between PD fallers and nonfallers [11]. Specifically, these studies have revealed a deterioration of the smoothness of trunk accelerations in people with PD as measured by harmonic ratios, which was more pronounced in those with a history of falls.

Despite emerging evidence that maintaining head stability during gait is a key determinant of postural control [12–16], accelerations of the head have been neglected in these previous studies examining upper body acceleration in PD. One potential reason head stability is important is that the head contains the visual and vestibular systems, which are critical for navigation and preplanning of adaptive motor strategies [13]. Head stability may have added importance for people with PD because they rely heavily on vision to maintain their postural control [5]. Recent evidence suggests that vision during gait is affected in PD [17] and that the smoothness of trunk accelerations is also altered [9, 10]. However, it has not yet been established whether PD affects the stability of the head during gait. A key mechanism in maintaining head stability is the attenuation of accelerations through the trunk. People with PD often develop axial rigidity, which may impair their ability to attenuate the accelerations that are applied to the lower limbs during gait from impacting on head stability. The measurement of attenuation of accelerations through the upper body has previously been investigated as a strong postural control indicator for children, adults, and elderly individuals [18–21] but has not yet been examined in people with PD.

The objective of the current investigation was to explore whether upper body accelerations obtained during gait provide sensitive proxy measures of postural control in people with Parkinson's disease (PD). More specifically, the aims of this study were to assess the magnitude, attenuation, and smoothness of upper body accelerations in people with PD compared to age-matched controls. We tested the hypotheses that: people with PD would demonstrate impaired smoothness and attenuation of accelerations. To address these aims, accelerations of the head, trunk, and pelvis were assessed during gait in a cohort of people with PD and an age-matched control group.

## 2. Materials and Methods

### 2.1. Participants

A subsection of community dwelling older adults and people with PD were tested as part of the ongoing ICICLE-PD (Incidence of Cognitive Impairment in Cohorts with Longitudinal Evaluation—Parkinson's Disease) Gait study [22, 23]. Participants were excluded if they had any neurological (other than PD), orthopaedic, or cardiothoracic conditions that may have markedly affected their walking or safety during the testing sessions. In addition, PD participants had to be diagnosed with idiopathic PD according to the UK Parkinson's Disease Brain Bank criteria and were excluded if they presented with significant memory impairment (Mini Mental State Exam (MMSE) ≤ 24 [24]), dementia with Lewy bodies, drug induced parkinsonism, “vascular” parkinsonism, progressive supranuclear palsy, multiple system atrophy, corticobasal degeneration, or poor command of English. This study was conducted according to the Declaration of Helsinki and had ethical approval from the Newcastle and North Tyneside research ethics committee. All participants signed an informed consent form.

### 2.2. Experimental Protocol

All participants walked for two minutes at their preferred pace around a 25 m circuit, fully described in [27]. Spatiotemporal gait variables (walking speed, step time, step length, and step width) were measured using a 7 m long Gaitrite pressure activated electronic walkway (Platinum model Gaitrite, software version 4.5, CIR systems, United States of America). Upper body accelerations were measured using three OPAL inertial sensors sampling at 128 Hz (APDM Inc, Portland, OR, USA) located at 5th lumbar vertebra to represent the pelvis level (P), the 7th cervical vertebra to represent the shoulder level (S) and upon the back of the head (H). The Gaitrite and the OPAL system were synchronised and the data was collected using the same A/D converter.

### 2.3. Data Analysis

To ensure only steady-state, straight-line walking was analysed, only the portion of the acceleration data recorded while participants who were in contact with the Gaitrite walkway was used. As detailed in Mazzà et al. [20], prior to collecting the gait data, a calibration trial was captured using a sensor placed on the floor to create a global reference frame for the laboratory. Thereafter, the local reference frame of each sensor was reoriented for each time sample to the newly established global reference frame [19, 28]. Following, the acceleration data was further segmented based upon the foot contact and foot off values obtained from the Gaitrite walkway. Then, the mean value of the signal was removed and a low-pass fourth order Butterworth filter with a cut-off frequency of 10 Hz was applied [19]. Data for each stride was normalised to 100 data points using linear interpolation. All signals were processed using MATLAB (version 8.1.0).

### 2.4. Magnitude of Acceleration

The magnitude of accelerations was calculated using the root mean square (RMS) of the accelerations, measured by each sensor for each stride in the Anteroposterior (AP), Mediolateral (ML), and Vertical (V) directions.

### 2.5. Attenuation of Acceleration

The ability to attenuate accelerations through the upper body was quantified using the attenuation coefficient. The attenuation coefficient describes the ability to reduce accelerations from inferior to the superior anatomical locations and was calculated using the RMS values for each stride.

The attenuation coefficients were computed using the RMS values of the head (RMS_H_), shoulder (RMS_S_), and pelvis (RMS_P_) as follows [18–20]:

(1) $$\begin{array}{c} C_{\text{PH}}=\left(1-\;\frac{{\text{RMS}}_{\text{H}}}{{\text{RMS}}_{\text{P}}}\right)\times 100,\\ C_{\text{PS}}=\left(1-\frac{{\text{RMS}}_{\text{S}}}{{\text{RMS}}_{\text{P}}}\right)\times 100,\\ C_{\text{SH}}=\left(1-\frac{{\text{RMS}}_{\text{H}}}{{\text{RMS}}_{\text{S}}}\right)\times 100 \end{array}$$

with *C*_PH_ representing the attenuation from the pelvis to the head, *C*_PS_ representing the attenuation from the pelvis to the shoulder, and *C*_SH_ representing the attenuation from the shoulder to the head. Each equation provides a percentage representing the amount of acceleration that is attenuated from the inferior sensors to the superiorly located sensor. A positive coefficient indicates reduced acceleration at the superiorly located sensor relative to the inferiorly located sensor. A negative coefficient value indicates a greater acceleration at the superiorly located sensor.

### 2.6. Smoothness of Accelerations

We quantified the smoothness of upper body accelerations using the harmonic ratio (HR). The HR accurately describes the step-to-step symmetry within a stride but for upper body gait analysis is also commonly referred to as a measure smoothness [29]. The HR was calculated via discrete Fourier transform for each of the acceleration components measured at the H, S, and P levels in the AP, ML, and V directions [30]. The fundamental frequency was set equal to the stride frequency.

*For the AP and V Components, the HR Was Defined as:*

(2) $$\begin{array}{c} \text{HR}=\frac{\Sigma \;\;\text{Amplitudes}\;\;\text{of}\;\;\text{even}\;\;\text{harmonics}}{\Sigma \;\;\text{Amplitudes}\;\;\text{of}\;\;\text{odd}\;\;\text{harmonics}} \end{array}$$

*For the ML Component, the HR Was Defined as:*

(3) $$\begin{array}{c} \text{HR}=\frac{\Sigma \;\;\text{Amplitudes}\;\;\text{of}\;\;\text{odd}\;\;\text{harmonics}}{\Sigma \;\;\text{Amplitudes}\;\;\text{of}\;\;\text{even}\;\;\text{harmonics}} \end{array}$$

Higher values of HR are associated with a higher similarity between the pattern of the upper body movements occurring during the right and left steps and are therefore favourable [9, 31]. Following calculation, the HR's were normalised to each participant's gait speed [9, 14].

### 2.7. Statistical Analysis

A series of two-tailed paired *t*-tests were used to test the difference between groups for the magnitude, attenuation, and smoothness of accelerations. The level of significance was set at *P* = 0.05. Given the exploratory nature of this study, the *P* value was not adjusted for multiple comparisons.

## 3. Results

The characteristics of the participants are reported in Table 1. All the participants with PD were tested within 18–54 months post diagnosis. No significant differences were found between the two groups in terms of anthropometric characteristics or spatiotemporal gait values.

### 3.1. Magnitude of Acceleration

Significantly higher ML head accelerations were observed in people with PD compared to controls (1.08 ± 0.29 m/s^2^ versus 0.86 ± 0.21 m/s^2^, *P* = 0.024) but not at the pelvis or the shoulder level. There were no other significant between-group differences although AP and V head accelerations tended to be greater in the PD group (Table 2).

### 3.2. Attenuation of Acceleration

**Figure 1:**
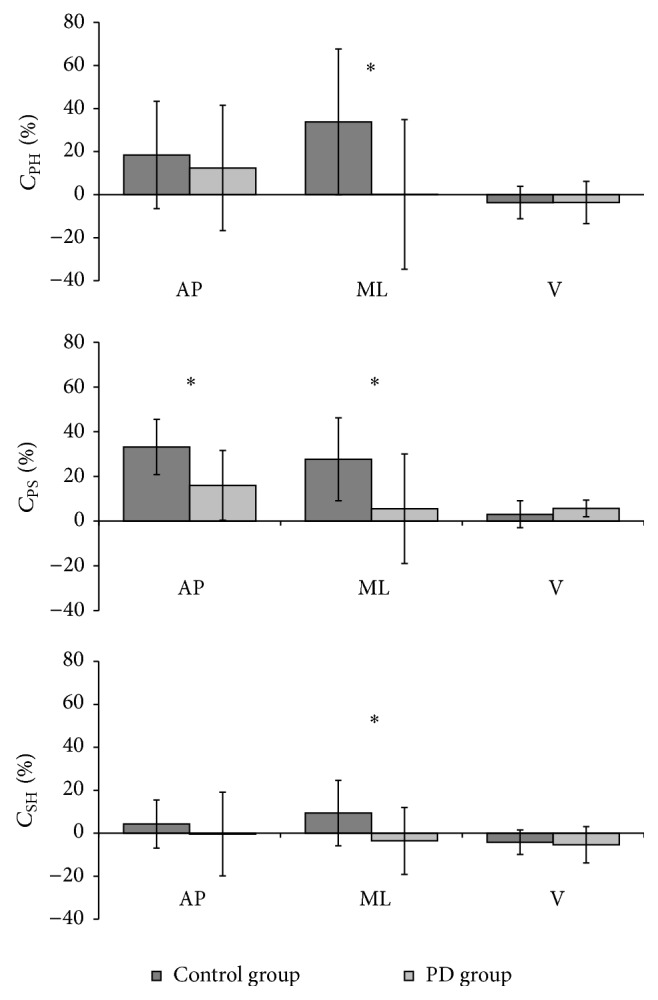
Mean (±SD) values of the attenuation coefficients (*C*_PH_, *C*_PS_, and *C*_SH_) of the three acceleration components (AP = anterior/posterior, ML = medial/lateral; V = vertical), computed for the control and group with PD. ^*^ *P* < 0.05.

People with PD did not attenuate AP or ML accelerations as well as controls (Figure 1). For *C*_PH_, a significant difference existed between PD and the control participants in the ML direction (0.12 ± 34.7% versus 33.8 ± 21.3%, *P* = 0.003). For *C*_PS_, a significant difference existed between PD and controls in the AP (16.0 ± 15.6% versus 33.1 ± 12.4%, *P* = 0.002), as well as the ML direction (5.5 ± 24.5% versus 27.7 ± 18.6%, *P* = 0.009). For *C*_SH_, a significant difference existed between the PD and the control group in the ML direction (−3.6 ± 15.5% versus 9.4 ± 15.3%, *P* = 0.031).

### 3.3. Harmonic Ratio

The HRs normalised to gait speed showed no significant differences between the PD and control participants (Table 3).

## 4. Discussion

Our current investigation provides evidence that upper body accelerations obtained during gait provide sensitive measures of postural control in people with Parkinson's disease (PD). As hypothesised, the results of this study showed that people with PD walked with altered upper body accelerations compared to age-matched controls. In particular, people with PD walked with greater magnitude of ML head accelerations and demonstrated impaired attenuation of accelerations from the pelvis and neck to the head. In contrast to our hypothesis, smoothness of upper body accelerations as measured by the HR was not significantly affected in this sample of PD.

To our knowledge, this is the first study to show impaired head stability in people with PD using inertial sensors. A greater magnitude of ML head acceleration was found for the PD group. This was interpreted as a result of poor postural control for the PD participants and a failure to stabilise their head in space [18, 21]. High values for the head accelerations have been previously described as a reduced ability to stabilise the head in space. This is particularly crucial for people with PD because of their aforementioned increased dependence upon visual input for correcting postural control [5]: higher accelerations are likely disturbing their visual system, leading to an impaired ability to preplan effective motor strategies [13], causing an increased likelihood to fall. Although they might be a useful measure of postural control, RMS values of head accelerations are known to be dependent upon step length and gait speed [31]. Despite no significant differences being observed for these parameters between the PD and the control group in this sample, it is common that PD affects both gait speed and step length [1–3]. As a result, the magnitude of accelerations may lack sensitivity when used for discriminating PD patients, which in other studies have been shown to possess a decreased step length and gait speed, when compared to age-matched controls.

Alternatively, being computed as a ratio between accelerations measured during the same trial [18], the coefficients of attenuation do not suffer from being speed dependent. In the current investigation; the coefficients of attenuation provided insight into why the PD participants demonstrated greater accelerations at the head. Participants with PD were less able to attenuate accelerations through the trunk, as shown by impaired pelvis-shoulder attenuation coefficients, which were reduced on average by at least a half in the PD cohort, both in the AP and in the ML direction. It is not possible to fully explain why the people with PD did not attenuate accelerations well through the upper body; however, it may be associated with* en bloc* movement and axial rigidity. It has previously been stated that increased rigidity may cause underlying changes in the physiological and mechanical functioning of the axial muscles which results in* en bloc* movement, where the head, trunk and pelvis move together as one rigid unit [12, 32]. It might be assumed that the same mechanisms could be responsible for poor attenuation of accelerations through the spine in PD. However, more research is certainly needed to test this hypothesis and explain the mechanisms ruling altered head accelerations and poor attenuation in PD, as well as the implications of poor head stability on vision and postural control.

Interestingly, the findings regarding attenuation coefficients were strongest in the ML direction. Similar results were found even when analysing healthy elderly subjects [21]. The fact that instability was predominantly found in the ML direction, suggests that when utilising a coefficient of attenuation, the ML direction is potentially most informative of an impaired walking stability. Consequently, assessments in the ML direction may be best for proxy measures of postural control in PD.

In contrast with our hypothesis and previous studies [9], the smoothness of upper body accelerations (harmonic ratios) was not significantly affected in our sample of PD participants. The discrepancy with previous studies is unlikely due to methodological differences, as the studies were similar in design. It is possible, however, that we were statistically underpowered to detect group difference, as suggested by a 25% reduction of AP HRs and 16% reduction of ML HRs at the head in the PD group that did not reach statistical significance (*P* = 0.106). Further research is required to determine the effectiveness of harmonic ratios as a sensitive measure to PD at different stages of their disease progression, as well as its ability to predict future falls.

Clinicians require objective measures to assess postural control during locomotion in people with PD to supplement standard clinical assessments and conventional rating scales which are not sensitive to subtle postural control disturbance [31, 33, 34]. Our findings indicate that it is feasible to measure the magnitude, attenuation, and smoothness of upper body accelerations in people with PD using body worn sensors. The rapid technological development of inertial sensors may afford a quick, clinically appropriate, and cost effective method to measure postural control in the clinic and community settings [5]. Specifically, the attenuation coefficient is a promising measure that is sensitive to PD; however, larger longitudinal studies are needed to assess its ability to monitor disease progression, determine intervention efficacy, and inform clinical management [5, 34, 35].

## 5. Conclusion

The current investigation suggests that assessing upper body acceleration offers additional and unique information about postural control during gait in people with PD. In particular, the magnitude of ML head accelerations and attenuation of upper body acceleration appear sensitive to PD and consequently hold promise as useful proxy measures that can be utilised in clinical and community settings.

## Figures and Tables

**Table 1:** The mean (±SD) participant characteristics and spatial-temporal gait variables for the PD and control group.

	PD (*n* = 13)	Control (*n* = 19)	*P* (*t*-test)
Age (years)	69.7 ± 11.1	70.2 ± 6.7	0.90
Height (m)	1.70 ± 0.10	1.72 ± 0.10	0.99
Mass (Kg)	77.9 ± 13.3	83.2 ± 14.2	0.30
BMI	26.1 ± 3.3	28.0 ± 4.5	0.20
MDS UPDRS III	35.6 ± 12.6	NA	NA
Hoehn and Yahr stage	HY II: 11; HY III: 2	NA	NA
Gait speed (m/s^2^)	1.22 ± 0.22	1.32 ± 0.15	0.14
Step time (s)	0.54 ± 0.21	0.54 ± 0.44	0.97
Step length (cm)	0.66 ± 0.12	0.71 ± 0.07	0.15
Step width (cm)	0.09 ± 0.03	0.09 ± 0.02	0.46

^*^Significant difference at *P* < 0.05.

BMI: body mass index.

MDS UPDRS III: Movement Disorders Society Revised Unified Parkinson's Disease Rating Scale–Movement Subsection [25].

HY: Hoehn and Yahr stage [26].

**Table 2:** The mean (±SD) root mean square (RMS) for the PD and the control participants calculated at the head (H), shoulder (S), and the pelvis (P) levels.

Sensor location	Component	PD	Control	*P* (*t*-test)
H	AP	1.02 ± 0.24	0.92 ± 0.20	0.22
	ML	1.08 ± 0.29	0.86 ± 0.21	0.02^*^
	V	2.15 ± 0.74	2.41 ± 0.47	0.26

S	AP	1.03 ± 0.18	0.96 ± 0.16	0.31
	ML	1.05 ± 0.24	0.96 ± 0.17	0.25
	V	2.04 ± 0.64	2.28 ± 0.46	0.24

P	AP	1.28 ± 0.38	1.47 ± 0.33	0.14
	ML	1.17 ± 0.36	1.41 ± 0.42	0.11
	V	2.16 ± 0.70	2.35 ± 0.47	0.37

^*^Significant difference at *P* < 0.05.

H: head; S: shoulder level; P: pelvis.

AP: anterior/posterior; ML: medial/lateral; V: vertical.

**Table 3:** The mean (±SD) Harmonic ratios normalised to gait speed for the PD and the control participants calculated at the head (H), shoulder (S), and the pelvis (P) levels.

Sensor location	Component	PD	Control	*P* (*t*-test)
H	AP	0.71 ± 0.36	0.53 ± 0.23	0.11
	ML	1.22 ± 0.56	1.02 ± 0.38	0.27
	V	2.03 ± 0.57	2.18 ± 0.60	0.50

S	AP	0.70 ± 0.23	0.66 ± 0.22	0.64
	ML	0.80 ± 0.50	0.80 ± 0.22	0.95
	V	2.34 ± 0.77	2.51 ± 0.72	0.55

P	AP	1.22 ± 0.38	1.13 ± 0.48	0.61
	ML	1.05 ± 0.69	0.80 ± 0.38	0.22
	V	2.02 ± 0.60	2.17 ± 2.17	0.58

H: head; S: shoulder level; P: pelvis.

AP: anterior/posterior; ML: medial/lateral; V: vertical.
